# Screening-based discovery of drug-like *O*-GlcNAcase inhibitor scaffolds

**DOI:** 10.1016/j.febslet.2009.12.020

**Published:** 2010-02-19

**Authors:** Helge C. Dorfmueller, Daan M.F. van Aalten

**Affiliations:** Division of Molecular Microbiology, College of Life Sciences, University of Dundee, Dundee DD1 5EH, Scotland, UK

**Keywords:** *O*-GlcNAc, Posttranslational modification, Inhibitor, Crystal structure

## Abstract

*O*-GlcNAcylation is an essential posttranslational modification in metazoa. Modulation of *O*-GlcNAc levels with small molecule inhibitors of *O*-GlcNAc hydrolase (OGA) is a useful strategy to probe the role of this modification in a range of cellular processes. Here we report the discovery of novel, low molecular weight and drug-like *O*-GlcNAcase inhibitor scaffolds by high-throughput screening. Kinetic and X-ray crystallographic analyses of the binding modes with human/bacterial *O*-GlcNAcases identify some of these as competitive inhibitors. Comparative kinetic experiments with the mechanistically related human lysosomal hexosaminidases reveal that three of the inhibitor scaffolds show selectivity towards human OGA. These scaffolds provide attractive starting points for the development of non-carbohydrate, drug-like OGA inhibitors.

## 1 Introduction

The posttranslational modification of intracellular proteins with *O*-linked *N*-acetylglucosamine (*O*-GlcNAc) was discovered 25 years ago by Torres and Hart [1], breaking the dogma that protein glycosylation was restricted to the endoplasmic reticulum, Golgi apparatus, cell surface and extracellular matrix [2]. *O*-GlcNAc modified proteins are found mostly in the nucleoplasm, cytoplasm and to some extent in the mitochondria, and are not further extended by glycosylation. Similar to protein phosphorylation, the *O*-GlcNAc modification can dynamically and inducibly be transferred to, and removed from, proteins, and has been shown to be involved in signalling processes [3–5]. The *O*-GlcNAc modification is carried out by an enzyme called *O*-GlcNAc transferase (OGT) that utilises the nucleotide sugar donor UDP-GlcNAc, produced through the hexosamine biosynthetic pathway, to exclusively modify serine/threonine residues. OGT was first discovered in human, rat and the nematode *Caenorhabditis elegans* [6,7], subsequently described in *Arabidopsis thaliana* [8] and very recently in *Giardia* and *Cryptosporidium parvum* [9]. The *O*-GlcNAc modified protein can be transformed back into its native state by *O*-GlcNAc hydrolysis by *O*-GlcNAcase (OGA). OGA has been characterised in human, mouse, rat, *Drosophila* and *C. elegans* and was shown to be encoded by a single gene [10–14]. In all metazoa, *O*-GlcNAc modified proteins have been found in all functional classes [15,16,5]. Dynamic interplay between protein *O*-GlcNAcylation and phosphorylation has been observed on a number of proteins [17]. Specific Ser/Thr residues show reciprocal, same-site occupancy by phosphorylation or *O*-GlcNAcylation (e.g. c-myc and oestrogen receptor-β or tau-protein) or alternatively adjacent Ser/Thr residues can be occupied by *O*-GlcNAcylation and phosphorylation (e.g. p53-protein) [17].

For the past decade PUGNAc and streptozotocin (STZ), both inhibitors of the human OGA (hOGA), have been extensively used to raise cellular *O*-GlcNAc levels to study the function of protein *O*-GlcNAcylation [18,19]. Recently, it was discovered that NAG-thiazoline, a transition state analogue, can also be used in cell-based assays to potently inhibit hOGA [20]. However, all three compounds have disadvantages. PUGNAc and NAG-thiazoline were originally identified as potent inhibitors of human lysosomal hexosaminidases (HexA/B), members of the glycoside hydrolase family 20 (GH20) [21,22]. Recent kinetic and structural data obtained from hOGA and bacterial homologues (GH84 family members) have revealed that GH84 and GH20 perform hydrolysis via substrate-assisted catalysis and the active site architecture is structurally conserved [23–25]. Thus it is not surprising that hOGA and HexA/B are equally potently inhibited by NAG-thiazoline and PUGNAc. PUGNAc is a promiscuous inhibitor, targeting multiple enzymes from functionally related glycoside hydrolase families, including retaining α- and β-glucosaminidases from GH89 and GH3, respectively [26,27]. Consequently, in cell-based studies PUGNAc shows off-target effects, as shown recently by Macauley et al. [28]. The authors also used a 600-fold selective thiazoline derivative (NButGT) to inhibit hOGA in 3T3-L1 adipocytes. Whilst both compounds increased cellular *O*-GlcNAcylation levels, only the non-selective PUGNAc caused insulin resistance in 3T3-L1 adipocytes [28]. STZ, another weak inhibitor of hOGA, carries a reactive *N*-methylnitrosourea moiety that has been shown to be toxic to eukaryotic cells by DNA alkylation [29–31] and nitric oxide release [32]. STZ was shown to be particularly toxic to pancreatic β cells that secrete insulin, and it has since been used extensively to create animal models of type I diabetes [33]. However, several studies have shown that STZ toxicity does not correlate with hOGA inhibition, suggesting that STZ-dependent β-cell toxicity occurs independent of hOGA inhibition [34–36,31].

hOGA has so far resisted production in pure recombinant form with yields required for protein crystallography. Two bacterial apparent OGA orthologues from *Clostridium perfringens* and *Bacteroides thetaiotaomicron* have been studied by protein crystallography to obtain insight in the active site and the catalytic mechanism [24,37,25]. Recently, a series of NAG-thiazoline derivatives, as well as GlcNAcstatins, a glucoimidazole-based class of inhibitors, were described to inhibit hOGA potently and selectively [28,38–40]. Crystal structures of the two bacterial OGAs in complex with these compounds have been used to identify and iteratively improve protein–ligand interactions. However, all currently known OGA inhibitors have in common a carbohydrate scaffold, that is inherently non-drug-like when the Lipinski rules are considered [41] and not synthetically easily accessible for further optimization by medicinal chemistry. Thus, identification of novel, more drug-like, and synthetically accessible inhibitors of the GH84 enzymes could facilitate further efforts towards the identification of potent, cell permeable and metabolically stable OGA inhibitors. Ideally, such compounds would be selective for GH84 enzymes versus GH20 enzymes or could easily be modified to improve selectivity towards hOGA. A possible approach to identify molecules with these properties is by high-throughput screening. Here we report the result of a screen, together with kinetic and structural studies of the hits, resulting in the discovery of novel, drug-like scaffolds that competitively inhibit hOGA.

## 2 Results and discussion

### 2.1 Identification of novel OGA inhibitors from a high-throughput screen

**Fig. 1:**
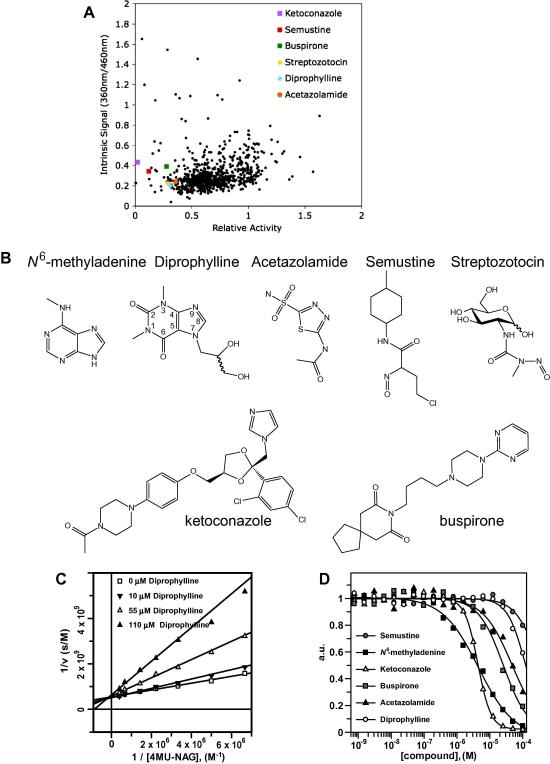
(A) Prestwick library screen against *Cp*OGA. The scatter-plot shows on the horizontal axis the remaining activity of *Cp*OGA in presence of the compounds (measured by the liberation of 4MU) and on the vertical axis the intrinsic absorbance/fluorescence of the compounds at the excitation and emission wavelength (360 nm/460 nm). Points in the lower left corner indicate compounds that do not absorb at the excitation wavelength, yet do exhibit reduced fluorescence and thus reduce *Cp*OGA activity. Six compounds are labelled in coloured dots, according to the legend in the graph. (B) Chemical structures of compounds selected with the aid of the Prestwick screening data: (a) *N*^6^-methyladenine, (b) diprophylline, (c) acetazolamide, (d) semustine, (e) streptozotocin (STZ), (f) ketoconazole and (g) buspirone. (C) Diprophylline is a competitive inhibitor of *Cp*OGA. Steady-state kinetic data (triplicates) using 0.2 nM *Cp*OGA, 0–25 μM substrate (4MU-GlcNAc), 0–110 μ inhibitor and 7 min reaction time were fitted using the standard equation for competitive inhibition in the GraFit program [52]. (D) Dose–response curves of the selected compounds against hOGA. Data obtained with the 4MU-GlcNAc assay (2 nM hOGA, 80 μM 4MU-GlcNAc, 0.7 nM–100 μM inhibitor, 60 min reaction time) from buspirone, diprophylline and acetazolamide, semustine, *N*^6^-methyladenine and ketoconazole were fitted using the standard IC_50_ equation in the GraFit program [52].

In order to identify new human *O*-GlcNAcase inhibitors, a high-throughput screening assay was performed based on the release of 4-methylumbelliferol (4MU) from the pseudo substrate 4MU-GlcNAc, initially using the bacterial OGA homologue from *C. perfringens* (*Cp*OGA) which has recently been shown to be a good model of hOGA [24]. The *Cp*OGA protein was screened against the library from Prestwick Chemicals Inc. that contains 880 off-patent small molecules. 85% of these compounds are currently marketed drugs. These compounds were dissolved in 100% dimethyl sulfoxide (DMSO) to generate library stocks with a final a concentration of 2 mg/ml. With an average molecular weight of 377 Da, screening assays were performed at an average concentration of 30.2 μM, with a final DMSO concentration of 1%. PUGNAc was used at a final concentration of 100 μM as a positive control. Probable false positives were identified by plotting the relative enzymatic activity against the intrinsic absorbance of the compound at the fluorescence excitation wavelength. After subtracting the intrinsic fluorescent signal from the compounds, the relative activity of *Cp*OGA in presence of the compounds in the screen was plotted against the real fluorescent signal from the reaction product (Fig. 1A). Surprisingly, a large number of compounds clustered around a relative activity of 0.6 (Fig. 1A) and not, as expected, around 1.0. This may be caused by the fact that those compounds are real inhibitors with much smaller molecular weights than the average 377 Da and were thus screened at effective concentrations higher than 30.2 μM. Only compounds that showed inhibition >60% at the assumed concentration of 30.2 μM (equal to a relative activity smaller than 0.4) were considered for further analysis and prioritised by the presence of chemical features likely to be compatible with the GH84 active site.

### 2.2 Selected screening hits competitively inhibit *Cp*OGA in the micromolar range

A number of compounds that inhibit *Cp*OGA at the assumed screening concentration of 30.2 μM to more than 60% were selected from the Prestwick screen hits (Fig. 1A). Streptozotocin (STZ), a known hOGA and *Cp*OGA inhibitor [31], was identified from the screen. The most potent compound was ketoconazole, followed by semustine (Fig. 1A). Buspirone, STZ and diprophylline show similar inhibition under the screening conditions, followed by acetazolamide (Fig. 1A). All these compounds were commercially available. 6-Methylaminopurine, a derivative of the weak (30% inhibition) hit 6-benzylaminopurine, was also purchased, given the recently published purine derivatives that inhibit chitinases, enzymes that are mechanistically related to OGA [42,43,24]. The molecular weights of the resulting selected compounds (Fig. 1B) were used to calculate the true screening concentration (Table 1). The compounds were then evaluated for *Cp*OGA inhibition using a dose–response experiment (Table 1). Ketoconazole appears to be the most potent *Cp*OGA inhibitor with an IC_50_ of 2 μM. *N*^6^-methyladenine inhibits with an IC_50_ of 21 μM, whilst semustine, buspirone and diprophylline inhibit with an IC_50_ of 39 μM, 50 μM and 60 μM, respectively. The dose–response curve of acetazolamide yields an IC_50_ of 80 μM. The ranking of the levels of inhibition of these compounds is the same from the screening and IC_50_ data.

Further kinetic experiments addressed the inhibition mode of the four most potent compounds (diprophylline, ketoconazole, semustine and *N*^6^-methyladenine) against *Cp*OGA. Steady state kinetics were measured to determine the inhibition mode and the corresponding inhibition constant, *K*_i_. Concentrations of the inhibitors were chosen according to the previously determined IC_50_ values (Table 1). For all four compounds, Lineweaver–Burk analysis indicated that the pseudo-substrate 4MU-GlcNAc and the compounds compete for binding to the same *Cp*OGA active site (Fig. 1C, Table 1). The absolute inhibition constants against *Cp*OGA measured for diprophylline (*K*_i_ = 25 μM), semustine (*K*_i_ = 23 μM) and *N*^6^-methyladenine (*K*_i_ = 14 μM) are in good agreement with the IC_50_ data (Table 1). Interestingly, *N*^6^-methyladenine is the most potent *Cp*OGA inhibitor identified, despite having the lowest molecular weight (Table 1).

### 2.3 *N*^6^-methyladenine is an efficient, selective, micromolar hOGA inhibitor

The objective of this study was to identify new chemical scaffolds that are inhibitors of hOGA. Thus, inhibition studies of the Prestwick screen-derived *Cp*OGA inhibitors (Fig. 1B) were carried out using recombinant hOGA protein (Fig. 1D). Both ketoconazole and *N*^6^-methyladenine inhibit hOGA with IC_50_ values of 4 μM (Table 1). Buspirone and acetazolamide inhibit hOGA with IC_50_ values of 25 and 47 μM, whilst diprophylline and semustine are weak hOGA inhibitors with inhibition constants of 120 and 260 μM.

Compared to the inhibition constants obtained against *Cp*OGA (Table 1), only the inhibition constant for semustine is significantly higher for hOGA than for *Cp*OGA. The IC_50_ values determined for acetazolamide, buspirone and ketoconazole against hOGA protein are in agreement with the inhibition constants determined for *Cp*OGA (Table 1). However, *N*^6^-methyladenine appears to more potently inhibit the human enzyme (IC_50_ = 4 μM for hOGA, vs. 21 μ for *Cp*OGA, Table 1). Since the active site architecture of *Cp*OGA and hOGA are closely related [24], it is assumed that the inhibitors bind to hOGA competitively, as determined for *Cp*OGA. Therefore, four compounds that competitively inhibit hOGA with an IC_50_ < 50 μM have been identified from screening the Prestwick Chemical library with the bacterial homologue *Cp*OGA.

Ligand efficiency is a powerful tool to evaluate the usefulness of a scaffold as a starting point for further medicinal chemistry [44–46]. The binding efficiency index (BEI) [46] was calculated for all drug-like *O*-GlcNAcase scaffolds and compared to the BEI of well known potent hOGA inhibitors NAG-thiazoline and PUGNAc (Table 1). Interestingly, while the small compound *N*^6^-methyladenine is a weaker inhibitor of hOGA than PUGNAc and NAG-thiazoline in absolute terms, in terms of ligand efficiency it, with a BEI of 34 (NAG-thiazoline and PUGNAc show a BEI of 32 and 21, respectively), is a more efficient binder.

To investigate whether the compounds identified from the Prestwick library screen display selectivity for hOGA over the closely related lysosomal hexosaminidases A/B (HexA/B), the enzymatic activity of HexA/B in the presence of each the compounds was analysed (Table 1). Ketoconazole, acetazolamide and buspirone were determined to have inhibition constants between IC_50_ = 10 μM and 50 μM (Table 1). Comparison of these inhibition constants with those determined for the hOGA enzyme shows no selectivity for the human *O*-GlcNAcase (Table 1). Similarly, dipro-phylline, with an inhibition constant of IC_50_ = 200  μM for HexA/B, is not selective for hOGA. Interestingly, a 75-fold selectivity is observed for *N*^6^-methyladenine (IC_50_ = 300 μM for HexA/B vs. 4 μM for hOGA, Table 1).

### 2.4 Diprophylline mimics substrate binding in the OGA active site

**Fig. 2:**
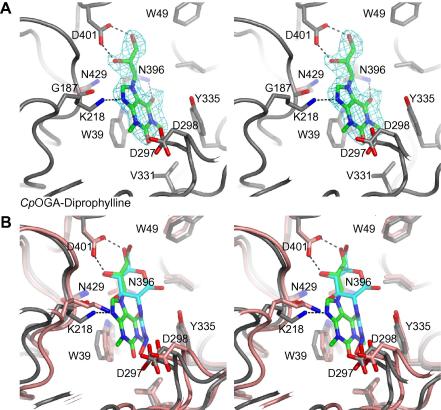
(A) Stereo figure of diprophylline in the active site of *Cp*OGA. The *Cp*OGA active site is shown with residues contributing to diprophylline binding and/or surrounding the compound, as sticks with grey carbon, red oxygen and blue nitrogen atoms. Diprophylline is depicted in sticks with green carbon, red oxygen, blue nitrogen atoms. Hydrogen bonds between the ligand and the protein are indicated by black dashed lines. Unbiased ∣*F*_*o*_∣ − ∣*F*_*C*_∣, *ϕ*_*calc*_ electron density (2.75 *σ*) for diprophylline is shown in cyan. (B) Stereo view of the superimposed crystallographically determined complexes of *Cp*OGA with diprophylline (colour scheme as in panel (A) and STZ (PDB entry 2VUR, depicted in sticks with cyan carbon, red oxygen and blue nitrogen atoms). Hydrogen bonds between the protein and diprophylline are indicated by black dashed lines.

To determine the binding mode of the competitive OGA inhibitors, these compounds were soaked into *Cp*OGA crystals. Ordered electron density could only be obtained for diprophylline. Diprophylline is a xanthine derivative that consists of two moieties. The xanthine moiety is methylated on the N1 and N3 ring atoms (Fig. 1B). Furthermore, the N7 atom is modified with a (2,3-dihydroxypropyl) moiety that possesses a stereo centre at the carbon of the secondary alcohol (Fig. 1B). The electron density for the inhibitor was located in the active site of *Cp*OGA, where it competes with substrate binding, in agreement with the competitive inhibition mode determined from kinetic studies. Diprophylline interacts with three active site residues via hydrogen bonds (Fig. 2A). The 2-OH and 3-OH hydroxyl groups from the 2,3-dihydroxypropyl moiety hydrogen bond to the carboxyl group of residue Asp401, which is conserved between bacterial (*Cp*OGA) and hOGA (Fig. 2A). This interaction mimics the interaction of GlcNAc 4/6-OH with Asp401 (Fig. 2A and B). The xanthine moiety is sandwiched between the two aromatic residues Trp394 and Tyr335, where it participates in *π*-stacking interactions (Fig. 2A). Furthermore, Lys218 and Asn396 hydrogen bond to the N9 and O6 atoms from the theophylline moiety, respectively (Fig. 2A). The electron density unambiguously represented the *S*-enantiomer of diprophylline (Fig. 2A).

The overall active site of the diprophylline-*Cp*OGA complex is in the same arrangement observed for the unliganded [24] and the STZ-*Cp*OGA complex [31] (Fig. 2B). Interestingly, the loop carrying the catalytic acid Asp298 and Asp297 is in the ‘open’ conformation. The peptide bond of Asp297–Asp298 is in the same conformation as described for the apo structure [24] and the STZ-liganded structure [31], thus the backbone carbonyl from Asp297 points into the active site and the side chain of Asp298 points out of the active site. Comparison of the STZ-*Cp*OGA complex with the diprophylline-*Cp*OGA complex reveals only minor differences in terms of the protein conformation. The loop carrying Asp297 and Asp298 has moved closer into the active site (maximum atomic shift = 1.2 Å), but neither Asp297 nor Asp298 interact with the compound. Superposition reveals that the O6 carbonyl group of diprophylline occupies the same position as the carbonyl oxygen of STZ (positional shift = 0.35 Å), and accepts a similar hydrogen bond from N*δ*2 of (the conserved) Asn396 at the back of the active site (Fig. 2A and B).

## 3 Concluding remarks

Screening a bacterial homologue of hOGA against a small commercial library from Prestwick Chemicals Inc. has resulted in the discovery of the first inhibitors of human *O*-GlcNAc that do not exhibit a sugar-like scaffold (Fig. 1B). Four of the these molecules competitively inhibit the GH84 enzymes. Interestingly, the small molecule *N*^6^-methyladenine, is a potent hOGA inhibitor (IC_50_ = 4 μM and shows 75-fold selectivity for the hOGA enzyme over the lysosomal hexosaminidases. This compound could be a lead for chemical modification to increase both the selectivity and potency of inhibition. Since no structural data was obtained, interactions of *N*^6^-methyladenine with the GH84 active site remain unknown. Interestingly, recent studies have described the binding of xanthine-like compounds to the active site of *Aspergillus fumigatus* chitinase 1 B (*Af*ChiB) [47] and a virtual screening-based approach that resulted in the synthesis of a derivative with micromolar inhibition [43]. A similar strategy could be applied to *N*^6^-methyladenine, which binds with a BEI of 34 to the hOGA active site.

Diprophylline, another xanthine-based molecule, was identified as a micromolar inhibitor for hOGA and the binding mode was structurally determined. Only the *S*-isoform of diprophylline binds to the GH84 active site and interacts with several residues conserved between hOGA and *Cp*OGA (Fig. 2A and B). Diprophylline is an interesting lead that could be further exploited by structure-based design to generate more potent derivatives that may inhibit hOGA in vivo.

In summary, this study shows that it is possible to identify hOGA inhibitors with scaffolds different from a sugar core, with promising properties in terms of synthetic accessibility, potency and selectivity. This will stimulate future work, both in terms of a medicinal chemistry exploration of these scaffolds, and the identification of more potent inhibitors by screening campaigns on larger libraries.

## 4 Materials and methods

### 4.1 Cloning, expression and purification

*Cp*OGA and hOGA protein were expressed and purified following the protocol described previously [24,39,31,40].

### 4.2 Determination of the *Cp*OGA-diprophylline complex structure

*Cp*OGA crystals were produced as described previously [24]. Precipitant was carefully removed and solid diprophylline was added straight to the drop. After 30 min the crystal was removed and cryo-protected in mother liquor containing 15% glycerol. Diffraction data were collected to 2.25 Å at the ESRF, Grenoble on ID14-3, and processed with the HKL suite [48], resulting in a data set with 99.9% completeness (100% in the highest resolution shell) with an overall *R*_merge_ of 0.071 (0.535 in the highest resolution shell). Refinement was initiated using a native *Cp*OGA structure (PDB-code 2CBI), immediately revealing well defined ∣*F*_*o*_∣ − ∣*F*_*c*_∣, *ϕ*_calc_ electron density for the inhibitor, which was built with the help of a structure and topology generated by PRODRG [49]. Further model building with COOT [50]) and refinement with REFMAC [51] then yielded the final model with good statistics (*R*, *R*_free_: 19.8, 24.7).

### 4.3 Inhibitor library screening

Purified *Cp*OGA protein was screened against a commercial library (Prestwick Chemicals Inc. France) containing 880 off-patent small molecules (85% of which are marketed drugs). The compounds were stored in 100% dimethyl sulfoxide (DMSO) at a concentration of 2 mg/ml – *Cp*OGA hydrolyses 4MU-GlcNAc without significant loss of activity at up to 4% DMSO. 0.5 μl aliquots of the compounds from the library were pipetted into 96 well-plates. 44.5 μl of the standard reaction mixture containing *Cp*OGA protein at a final concentration of 0.2 nM (in 50 μl final reaction volume) was added to the compounds. 5 μl of the fluorescent substrate 4MU-NAG was added in a 10-fold concentration (32 μM) to initiate the reaction after a 5 min incubation time of the *Cp*OGA enzyme with the compound. The reaction was stopped after 7 min at RT (20 °C) using standard procedure and the fluorescent signal was measured using the standard procedure described previously [24,31,39,40]. Hits were selected using several criteria: the compounds had to inhibit *Cp*OGA greater than 60% at the concentration screened and to posses a chemical scaffold with chemical features compatible with binding to the active site of GH84 enzymes.

### 4.4 Inhibition measurements of *Cp*OGA, hOGA and human HexA/B

Further kinetic experiments to determine the mode of inhibition were carried out according to the procedure described previously [40]. Ketoconazole, acetazolamide, buspirone, diprophylline, *N*^6^-methyladenine, streptozotocin and semustine were purchased from Sigma. IC_50_ measurements with *Cp*OGA, hOGA and a mixture of human hexosaminidase A/B activities (Sigma A6152) against the compounds were performed using the fluorogenic 4MU-NAG substrate and standard reaction mixtures as described previously with some changes [39,31,40]. Standard reaction mixtures (50 μl) contained 0.2 nM *Cp*OGA, 2 nM hOGA or 50 μ units unit/ml HexA/B in McIlvaine buffer (0.2 M Na_2_HPO_4_ mixed with 0.1 M citric acid to pH 5.7) supplemented with 0.1 mg/ml BSA. IC_50_ determinations were carried out using substrate concentrations corresponding to the *K*_m_ established for *Cp*OGA (2.9 μM), hOGA (80 μM) and HexA/B (230 μM). The reactions were run at room temperature for 7 min (*Cp*OGA), 60 min (hOGA) or 15 min (HexA/B). Determination of the *K*_i_ for *Cp*OGA was performed by steady-state kinetics using the parameters described for *Cp*OGA IC_50_ determinations with a series of inhibitor concentrations (0–110 μM. The reactions were stopped by the addition of 100 μl 3 M glycine–NaOH, pH 10.3. The fluorescence of the released 4MU was quantified as described previously [39,31,40]. *K*_i_ experiments were performed in triplicate and IC_50_ determinations with single data points for a series of 18 concentrations between 0.7 nM and 100 μM.

## Figures and Tables

**Table 1:** Screening data, inhibition constants and binding efficiency obtained for the selected compounds.

Compound	MW (Da)	Screening (μM)	*Cp*OGA IC_50_ (μM)	*Cp*OGA *K*_i_ (μM)	hOGA IC_50_ (μM)	HexA/B IC_50_ (μM)	Selectivity (HexA/B/hOGA)	hOGA BEI^a^^,^^b^
Acetazolamide	222	90	80 ± 14	n.d.	47.0 ± 0.1	32 ± 2	0.7	21
Buspirone	386	52	50 ± 15	n.d.	25.0 ± 0.1	47 ± 3	1.9	13
Diprophylline	254	79	60 ± 10	25 ± 2	121 ± 10	200 ± 30	1.6	16
Ketoconazole	531	38	2.0 ± 0.4	15 ± 5	4.3 ± 0.4	11 ± 1	2.6	11
Semustine	248	81	39 ± 6	23 ± 7	262 ± 6	2500	9	16
*N*^6^-methyladenine	149	–	21 ± 4	14 ± 1	4.0 ± 0.3	300	75	34
Streptozotocin	265	87	30^c^	n.d.	1500^d^	45 000	30	11
NAG-thiazoline	219	–	–	n.d.	0.07^d^	0.07^d^	1.0	32
PUGNAc	353	–	–	0.005^e^	0.05^d^	0.05^d^	1.0	21

a BEI Binding Efficiency Index (BEI) [46] calculated for hOGA inhibition, BEI = −log(*K*_i_)/*M*,with *M* being the mass of the compound in kDa.

b The Cheng–Prusoff equation (*K*_i_ =  IC_50_/1 + ([*S*]/*K*_m_)) was used to convert the IC_50_ values to anabsolute inhibition constant (*K*_i_).

c Data taken from [31].

d *K*_i_ value from [20].

e *K*_i_ value from [24].
